# Parameters Identification for Photovoltaic Module Based on an Improved Artificial Fish Swarm Algorithm

**DOI:** 10.1155/2014/859239

**Published:** 2014-08-27

**Authors:** Wei Han, Hong-Hua Wang, Ling Chen

**Affiliations:** College of Energy and Electrical Engineering, Hohai University, Nanjing 211100, China

## Abstract

A precise mathematical model plays a pivotal role in the simulation, evaluation, and optimization of photovoltaic (PV) power systems. Different from the traditional linear model, the model of PV module has the features of nonlinearity and multiparameters. Since conventional methods are incapable of identifying the parameters of PV module, an excellent optimization algorithm is required. Artificial fish swarm algorithm (AFSA), originally inspired by the simulation of collective behavior of real fish swarms, is proposed to fast and accurately extract the parameters of PV module. In addition to the regular operation, a mutation operator (MO) is designed to enhance the searching performance of the algorithm. The feasibility of the proposed method is demonstrated by various parameters of PV module under different environmental conditions, and the testing results are compared with other studied methods in terms of final solutions and computational time. The simulation results show that the proposed method is capable of obtaining higher parameters identification precision.

## 1. Introduction

Due to the increasing price of fossil fuels and their possible depletion, the growing trend to use renewable energy sources has gained attention in recent years [1]. Solar energy is one of the most practical renewable energy sources, which has been used all over the world to provide an inexhaustible supply of energy, without pollution or global warming [2]. The solar energy is the fastest growing power-generation technology with an annual growth rate of 50% between 2005 and 2013, according to reports.

It is well known that numerous solar cells are connected in series and parallel to form a PV module. Accurate solar cell modeling has aroused sufficient attention over the past years. The output curves of a solar cell exhibit nonlinear and multivariable characteristics determined by the solar cell parameters that describe its model under different operating conditions [3, 4]. However, several models have been introduced and proved to be successful in representing the behavior of the solar cell systems by considering many physical variables. Among them, two equivalent solar cell models are widely used in practice: double and single diode models [5]. The double diode model contained seven unknown parameters and offers higher accuracy. On the contrary, the single diode model has five unknown parameters, so it is much more common to use. In fact, both double and single diode models require the knowledge of all unknown parameters, which is usually not provided by manufactures. The main parameters of both models comprise the photogenerated current (*I*_ph_), saturation current (*I*_*D*_), series resistance (*R*_*s*_), shunt resistance (*R*_sh_), and diode ideality factor (*n*). Precise parameters of mathematical model play a key role in the simulation, evaluation, and optimization of solar cell systems. As a result, it is necessary to take into consideration the parameters identification with a feasible optimization method.

During the last two decades, many methods for parameters identification of solar cells have been proposed. There are two main methods used in the literatures to solve the parameters identification for solar cell models: the traditional and intelligent optimization methods. Newton's method is commonly used in identifying the parameters by mathematical equations [6–9]. Despite having the advantage of simplicity, its drawback heavily depends on the selection of initial value. Furthermore, a new analytical solution method based on Lambert W-function has been proposed to estimate parameters of solar cell model [10, 11]. However, this method has limited scope of application and falls into local minimum point easily, which is the common deficiency of similar analytical methods. In addition, the parameters identification of solar cell model has the characters of being nonlinear, multivariable, and multimodal; the traditional optimization methods could not solve the parameter identification problem effectively. Based on the above discussion, the intelligent optimization methods have been proposed to gain the best solution of parameters identification for solar cell model recently, such as genetic algorithm (GA), particle swarm optimization (PSO), pattern search (PS), harmony search (HS), simulated annealing (SA), and artificial bee swarm optimization (ABSO). Though these intelligent algorithms outperform the traditional ones for solar cell parameters identification, they have their respective limitations. The parameters identification for solar cell model based on GA has the relatively high percentage of errors associated with the estimated parameters and the complex binary conversion belonging to GA implementation in [12]. In PSO, the selection of its inherent parameters is concerned with the balance between exploration and exploitation of particles, which may lead to trap into local optima [13, 14]. The PS performance presented by AlHajri et al. [15] depends strongly on the well-defined objective function and the computation time of PS optimization algorithm could not be guaranteed at a reasonable time. The diversity of HS algorithm could not be maintained to avoid premature convergence, unless some of the worse harmonies are well considered by a given probability [16]. El-Naggar et al. [17] proposed that the performance of SA is sensitive to the initial solution and difficult to determine the parameters of the cooling schedule through strict theoretical proofs. In [18], ABSO is sensitive in initial parameters setting and selected by roulette way, which results in diversity decreasing and individual prematurity. Therefore, the nonlinearity and multidimensionality of the solar cell characteristics expect a high-performance optimization algorithm.

Motivated by the behaviors of fish swarm, a novel heuristics called artificial fish swarm algorithm (AFSA) is introduced into this topic for the first time [19]. AFSA has many advantages, including good global convergence, strong robustness, insensitivity to initial values, and simplicity in implementing [20]. The proposed AFSA has been widely used in many different applications such as parameters analysis, neural network classifiers, signal processing, network combinatorial optimization, and complex function optimization [21–25]. Hence, this paper uses the AFSA to find the optimal parameters of solar cell model. Different from the standard AFSA operations, an intelligent mutation operator (MO) is employed to further promote the optimization performance of the algorithm in the later period of convergence process. To verify the advantages of the improved AFSA method, the proposed method is tested and applied to compare with other parameters identification methods by experimental voltage-current (*V*-*I*) data. The simulation results demonstrate that the improved AFSA is superior to other methods in terms of objective function with the minimum root mean square error (RMSE), computational time, and parameters identification precision.

The rest of this paper is arranged as follows.  Section 2 provides a description of both solar cell models used in this work.  Section 3 proposes the improved AFSA with MO (MAFSA) and applies it to the parameters identification for solar cell models. In addition, simulation results and discussion are presented in Section 4. Finally, Section 5 draws the conclusion.

## 2. PV Module Modeling and Problem Formulation

An accurate mathematical model describing the electrical characteristics of solar cell is needed in advance. So far, there are several equivalent circuit models which are proposed to simulate voltage-current (*V*-*I*) output curves of solar cells. Among them, two models are practically used, namely, the double and single diode models [26, 27]. These two models will be briefly introduced in the following subsections.

### 2.1. Double Diode Model

**Figure 1:**
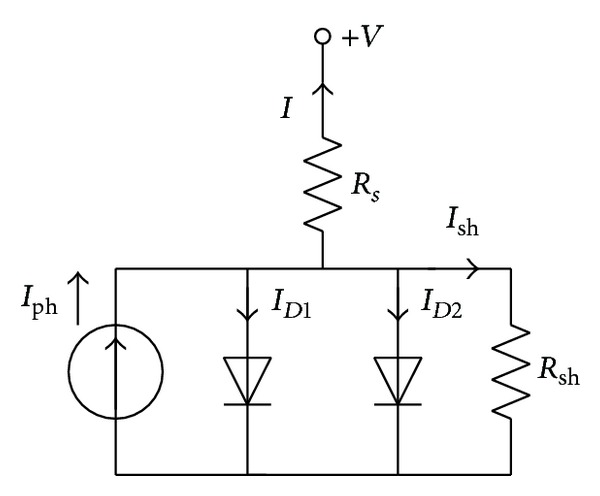
Equivalent circuit of a double diode model.

Generally speaking, an ideal solar cell model under illumination is a photogenerated current source connected in parallel with a rectifying diode. Nevertheless, the fact that the current source is also shunted by another diode modeled the space charge recombination current and a shunt leakage resistor to take into account the partial short circuit path near the cell's edges because of the semiconductor impurities and nonidealities. Moreover, a resistor is connected in series with the cell shunt elements due to the solar cell metal contacts and the semiconductor material bulk resistance. The equivalent circuit of this double diode model is shown in Figure 1.

In this double diode model, the terminal current of solar cell *I* can be expressed as follows:

(1) $$\begin{array}{c} I=I_{\text{ph}}-I_{D1}-I_{D2}-I_{\text{sh}}, \end{array}$$

where *I*_ph_ is the photogenerated current; *I*_*D*1_ and *I*_*D*2_ denote the first and second diode currents, respectively; *I*_sh_ is the shunt resistor current.

Taking the Shockley equation into consideration, the two related diode currents are illustrated in (2) and (3), respectively. In the meanwhile, the leakage resistor current *I*_sh_ is calculated as shown in (4):

(2) $$\begin{array}{c} I_{D1}=I_{\text{SD}1}\left[\exp\left(\frac{q\left(V+IR_{s}\right)}{n_{1}kT}\right)-1\right], \end{array}$$

(3) $$\begin{array}{c} I_{D2}=I_{\text{SD}2}\left[\exp\left(\frac{q\left(V+IR_{s}\right)}{n_{2}kT}\right)-1\right], \end{array}$$

(4) $$\begin{array}{c} I_{\text{sh}}=\frac{V+IR_{s}}{R_{\text{sh}}}, \end{array}$$

where *I*_SD1_ and *I*_SD2_ are the diffusion and saturation currents, respectively; *q* is the electronic charge; *V*_*L*_ denotes the terminal voltage; *R*_*s*_ and *R*_sh_ are the series and shunt resistances, respectively; *n*_1_ and *n*_2_ are the diffusion and recombination diode ideality factors, respectively; *k* is the Boltzmann constant; *T*(*K*) denotes the solar cell absolute temperature in Kelvin.

Substituting (2)–(4) into (1), the terminal current of solar cell in (1) is rewritten as shown in the following equation:

(5) I=Iph−ISD1[exp⁡(q(V+IRs)n1kT)−1]−ISD2[exp⁡(q(V+IRs)n2kT)−1]−(V+IRsRsh).

As can be seen, there are seven unknown parameters to be estimated for such a solar cell model, namely, *I*_ph_, *R*_*s*_, *R*_sh_, *I*_SD1_, *I*_SD2_, *n*_1_, and *n*_2_. To reflect the solar cell performance as well as that of the real system, it is essential to obtain an accurate parameters' identification that presents the characteristics of the solar cell.

### 2.2. Single Diode Model

**Figure 2:**
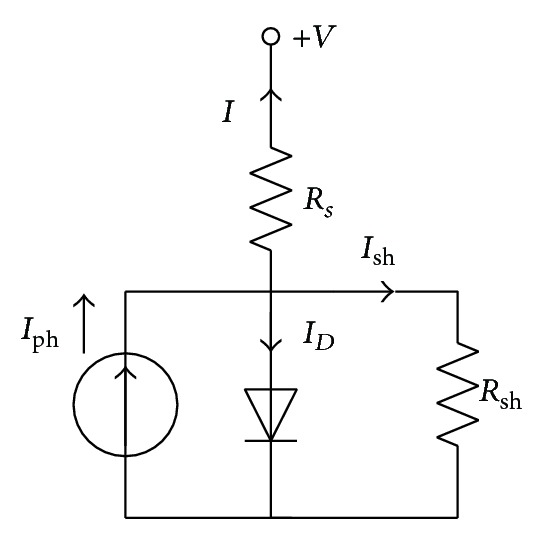
Equivalent circuit of a single diode model.

Due to the simplicity and accuracy, the single diode model is also used widely to represent the solar cell behavior. The concept of this model is inspired by combining together both diode currents, under the introduction of a nonphysical diode ideality factor *n*. In recent years, it has been validated that the single diode model can fit the experimental data successfully to some extent. The equivalent circuit of this single diode model is shown in Figure 2.

The representation of this model can be formulated as follows:

(6) $$\begin{array}{c} I=I_{\text{ph}}-I_{\text{SD}}\left[\exp\left(\frac{q\left(V+IR_{s}\right)}{nkT}\right)-1\right]-\frac{V+IR_{s}}{R_{\text{sh}}}. \end{array}$$

In this model, there are five unknown parameters to be identified, namely, *I*_ph_, *R*_*s*_, *R*_sh_, *I*_SD_, and *n*.

In conclusion, the double diode model significantly improves the accuracy but at the expense of additional parameter calculation. The single diode model is known to have a reasonable tradeoff between simplicity and accuracy under normal weather conditions. Therefore, this paper employs the single diode model to identify the parameters of PV module.

### 2.3. PV Module Model

The PV module is formed in the way that parallel connection arrays consisting of solar cells are first connected in series and then connected in parallel. A blocking diode is connected in series with each PV string to prevent excess current produced by other strings from flowing back in a failed string. In series strings, a bypass diode is connected across each PV module or number modules, providing energy releasing route to prevent power mismatching losses even when partially shaded or soiled. Furthermore, the mathematical expression of the terminal equation related to the currents and voltages of a PV module with *N*_*P*_ parallel strings and *N*_*S*_ series cells is shown in

(7) I=IphNp−ISDNp[exp⁡(qVNp+qIRsNsnkTNsNp)−1]−VNp+NsIRsNsRsh.

However, for the PV module, it should be noted that the identified parameters show a good association with the experimental data; they cannot be exactly related to the physical phenomena due to the differences between the solar cells connected to form the modules.

### 2.4. Problem Formulation

It is obviously noted that (7) is implicit and nonlinear transcendental functions, including the total output current produced by the PV module in each side of the equations. Moreover, these parameters such as *R*_*s*_, *R*_sh_, *I*_ph_, *I*_SD_, and *n* vary with temperature, solar irradiance, and explicit analytical solutions for either output current *I* or voltage *V*. This kind of equations is often solved by numerical methods, curve fitting techniques, and intelligent optimization methods [28]. Based on the characters of parameters identification for PV module, this paper proposes the AFSA optimization method to obtain the accurate parameters by minimizing a predetermined objective function.

### 2.5. Objective Function

In order to extract the parameters of PV module by the collected* V*-*I* data, an objective function should be established before making an optimization process [29]. In this paper, the RMSE is proposed as the objective function to quantify the difference between the calculated results and the measured data. According to the above discussion, the relationship between the output current and voltage described in (7) should be rewritten in the homogeneous forms as follows:

(8) f(Va,Ia,Iph,ISD,Rs,Rsh,n)  =Ia−IphNp  +ISDNp[exp⁡(q(VaNp+RsIaNs)nkTNpNs)−1]  +VaNp+NsRsIaNsRsh,

where *V*_*a*_ and *I*_*a*_ are the voltage and current in actual operation, respectively.

Moreover, the new objective function that sums RMSE for any given set of measurements is defined as

(9) $$\begin{array}{c} \text{RMSE}=\sqrt{\frac{1}{M}\overset{M}{\underset{i=1}{\sum }}{\left(f_{i}\left(V_{a},I_{a},x\right)\right)}^{2}}, \end{array}$$

where *M* is the number of the measured results; *x* = [*I*_ph_, *R*_*s*_, *R*_sh_, *I*_SD_, *n*] is the vector of parameters to be identified.

In this paper, the objective function expressed in (9) is utilized to provide a guidance for identifying different model parameters. Thus, a different objective is presented as an alternative one to find the optimal sets of solar cell parameters.

During the process of AFSA optimization, the objective function is introduced to be minimized with respect to the parameter ranges. The upper and lower boundaries of each parameter, provided by the literature survey, are tabulated in Table 1.

In theory, the accurate values of unknown parameters are obtained, along with the objective function which will be close to zero. That is to say, in order to make the simulation results better fit the actual values, the objective function in (9) needs to be minimized. Obviously, the smaller the objective function, the better the optimized parameters.

## 3. Solution to Solar Cell Models Based on an Improved AFSA

According to the above analysis, the parameters identification for solar cell models belongs to a class of NP-hard problems and also is very difficult to solve. Artificial fish swarm algorithm (AFSA), a novel intelligent algorithm, was first proposed in 2003 [19, 20]. It has potential to be one of the excellent techniques to obtain optimal or near-optimal solutions to the solar cell parameters identification problems.

### 3.1. Artificial Fish Swarm Algorithm

In a water area, fish are most likely distributed around the region where foods are the most abundant. A fish swarm completes its food foraging process by taking several simple social behaviors. It is found that there are three most common fish behaviors: (1) searching behavior, that is, fish tend to head towards food; (2) swarming behavior, gregarious fish tend to concentrate towards each other while avoiding overcrowding; (3) following behavior, the behavior of chasing the nearest buddy. Inspired by swarm intelligence, AFSA is an artificial intelligent algorithm based on the simulation of collective behavior of real fish swarms. It simulates the behavior of a single artificial fish (AF) and then constructs a swarm of AF. Each AF will search its own local optimum, pass on information in its self-organized system, and finally achieve the global optimum.

Suppose that the searching space is D-dimensional and there are *N* fish in the colony. The current state of an AF is a vector *X* = (*x*_1_, *x*_2_, …, *x*_*n*_), where *x*_*i*_  (*i* = 1, 2, …, *n*) is the variable to be optimized. The food consistence of AF in the current position is represented by *Y* = *f*(*X*), where *Y* is the objective function. The distance *D*_*ij*_ between *X*_*i*_ and *X*_*j*_ of individual AF can be expressed as ||*X*_*j*_ − *X*_*i*_||. *δ* is defined as the factor of crowded degree, which represents the crowded degree of a location nearby and avoids more AF to gather together.* Visual* is the perception scope of an AF, which determines the moving direction of each AF. When the perception range of* Visual* becomes larger, the observation of an AF can be more comprehensive. However, the number of fish should grow more so as to reach the calculation amounts. Under the actual situation, the appropriate value should be selected by the specific case.* Step* is the largest moving step of an AF. For fear of missing the optimum solution, the length of* Step* should not be set too large. Of course, the length of* Step* is too small to converge.* Try_number* is represented as the number of the biggest trial in the searching behavior.

**Figure 3:**
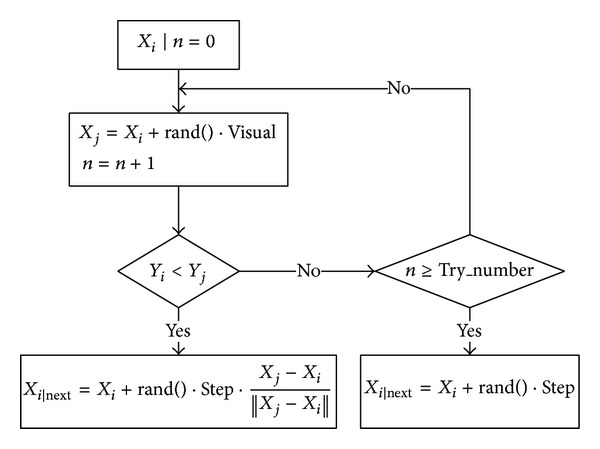
Searching behavior of AFSA.

**Figure 4:**
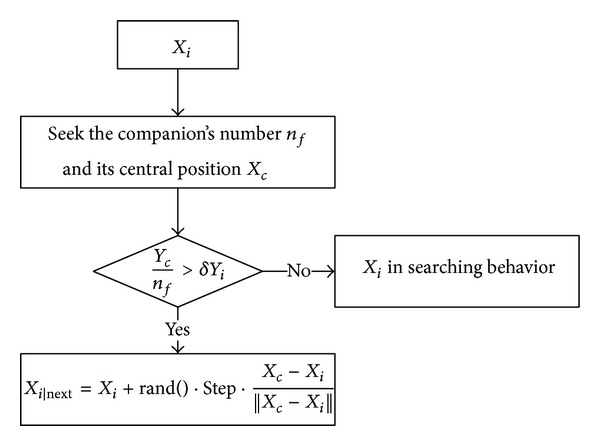
Swarming behavior of AFSA.

**Figure 5:**
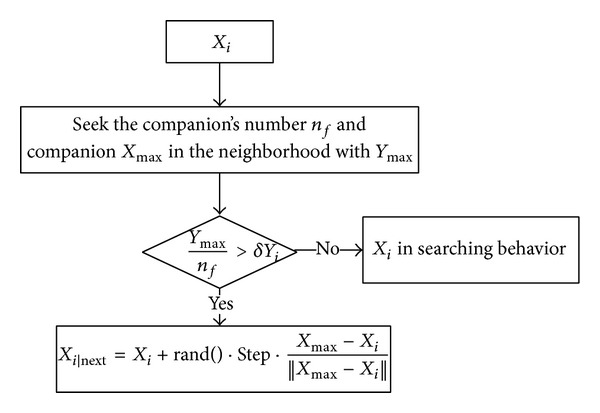
Following behavior of AFSA.

In the initial state of the algorithm, the variable of trial number should be defined as the trial times of AF searching for food. Then, the following steps are described the fish swarm behaviors.

(*1) Searching Behavior.* The fish follows the direction of food with high concentration when the fish is in the searching behavior. Suppose the current state of an AF is *X*_*i*_ and its fitness is *Y*_*i*_. A new state is randomly selected in its* Visual* field. If, in the maximum problem, *Y*_*i*_ < *Y*_*j*_ (as the maximum problem and minimum problem can be converted with each other, the maximum problem is discussed as an example in the following analysis), move a step in that direction; otherwise, select a state randomly again and judge whether it satisfies the aforementioned condition. If it cannot be satisfied after a predetermined times of* Try_number*, it moves a step randomly. The searching behavior is shown in Figure 3.

(*2) Swarming Behavior*. Swarming behavior is a behavior in which a fish swims towards the central position of swarm and avoids overcrowding. An AF at current state *X*_*i*_ seeks the companion's number *n*_*f*_ and its central position in its current neighborhood (*D*_*ij*_ < *Visual*); if *Y*_*c*_/*n*_*f*_ > *δY*_*i*_, it means that, at the center of the fish colony, there is enough food and it is not too crowded. The swarming behavior is illustrated in Figure 4.

(*3) Following Behavior.* Following behavior is the process where the fish captures the most active individual of swarm nearby. Suppose *X*_*i*_ is the current state of AF searching companion *X*_max⁡_ in the neighborhood with *Y*_max⁡_; if *Y*_max⁡_/*n*_*f*_ > *δY*_*i*_, this means that the current position of companion *X*_max⁡_ has a higher food consistence and it is not crowded enough. The AF will move a step towards the companion *X*_max⁡_; otherwise, it will continue the searching behavior. The following behavior can be seen in Figure 5.

(*4) Random Behavior.* The realization of the random behavior is relatively simple. It means selecting a state randomly in the* Visual* field and swimming towards that direction. In fact, that is a default behavior of the searching behavior; namely, *X*_*i*|next_ is the next position of *X*_*i*_:

(10) $$\begin{array}{c} X_{i|\text{next}}=X_{i}+r\cdot Visual, \end{array}$$

where *r* is the random number in range of [−1,1].

(*5) Bulletin*. Bulletin is used to record the AF's optimal state and the optimal value of the problem. Each AF updates its own state and compares it with the bulletin after making movements. If its current state of AF is better, then the value on the bulletin will be replaced. At the end, the value on the bulletin is the optimal solution of the problem.

In the process of AFSA, searching behavior lays the foundation for the AF; swarming behavior enhances the convergence of stability; following behavior ensures the convergence of quickness; behavior selection guarantees the high efficiency and stability of the algorithm. Through the behavior selection, the AFSA can form an optimization strategy with high efficiency. The procedure of the AFSA is shown in Algorithm 1.

### 3.2. AFSA with Added Mutation Operator

The AFSA has the ability to grasp the searching direction and avoid falling into the local optimum. But when some fish move in aimless randomly or gather around the local optima, the convergence speed will be slowed down greatly and the searching accuracy is greatly reduced. To avoid premature convergence, an intelligent MO similar to the genetic algorithm is employed to enhance the ability escaping from the local optima in this paper [12]. If the state of AF is not improved during the iterations and the AF has entered into a state of partial mining, it needs to be mutated. The specific steps are as follows.

1. Randomly select one of the variables in the position to plus one and choose the nonnull to minus one.
2. If the value of state is better than that of the current state, update the state of position; otherwise, go back to step (1) until the initial number of the mutating operation is satisfied.

By adding the mutation mechanism into the standard AFSA, it achieves the aim of altering the state of each AF. Through adjusting the swarms, the rate of convergence and the global searching ability of AFSA are both improved. The selection of mutating probability will have a great influence on the performance of the proposed algorithm, which has a positive correlation with the elapsed time. According to the experimental experience, the probability of mutation operator (PMO) selected as 1/(30*D*) ~ 1/(10*D*) (*D* is the dimension) can obtain a good performance. Usually, the PMO of an AF is assumed to be 0.03~0.1.

### 3.3. Identifying Parameters of Solar Cell Models with Improved AFSA

The steps of the proposed algorithm used in this work to obtain the optimal parameters of solar cell models can be summarized as follows.

**Step 1 .**

Initialize the state of fish swarm, such as* MAXGEN*,* Sizepop*,* Step*,* Try_number*,* Visual*, *δ*, and PMO.

**Step 2 .**

The values of the objective function for all individuals are evaluated, and the best individual is assigned to the bulletin board.

**Step 3 .**

The state of each fish is updated by the behaviors of searching, swarming, and following. A new fish swarm is generated.

**Step 4 .**

All the individuals are reevaluated. If one individual is superior to the bulletin board, it would replace the individual on the bulletin board.

**Step 5 .**

Add the mutation operator to the standard AFSA.

**Step 6 .**

Evaluate objective function and refresh the bulletin board.

**Step 7 .**

Steps 3 to 6 are repeated until the termination condition of the AFSA is met.

**Step 8 .**

The best individual of the fish swarm is selected as the optimal solution to the solar cell parameters identification.

**Figure 6:**
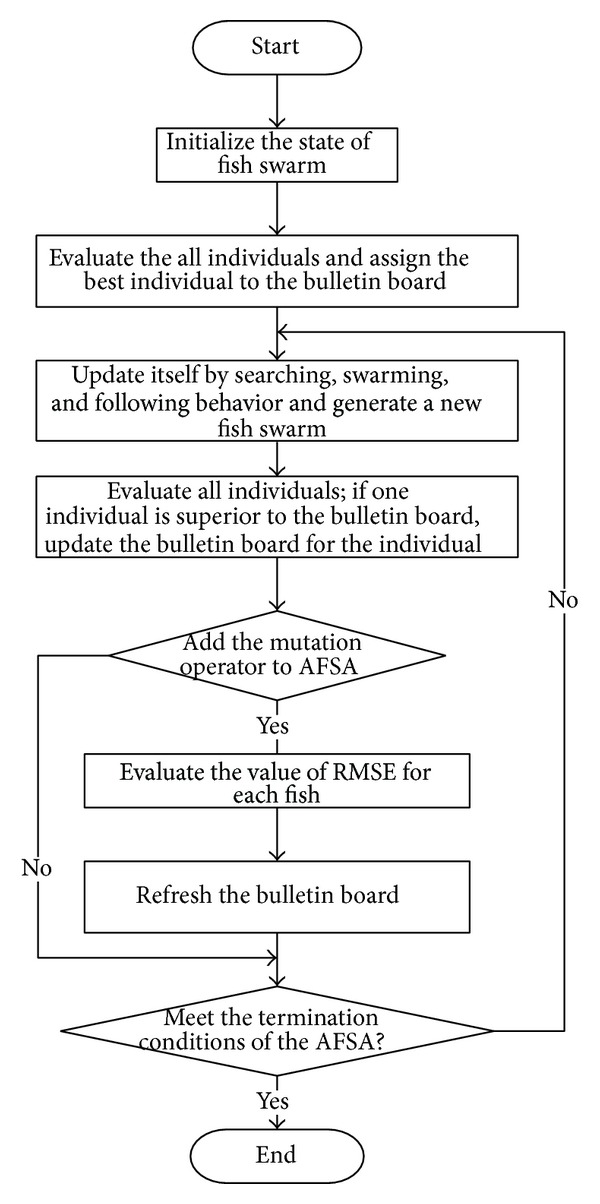
Flowchart of identifying PV module parameters with the improved AFSA.

In conclusion, the flowchart of identifying the PV module parameters is shown in Figure 6.

## 4. Results and Discussions

The improved AFSA technique is proposed to identify the parameters of PV module in this section. The efficiency of the improved AFSA-based parameters identification method is verified by identifying the experimental data of PV module under different irradiance and temperature conditions. Comparisons with other optimization algorithms for identification are also presented for the experimental data, which is generated using the single diode PV module model.

Since we cannot practically guarantee optimal parameters for the experimental PV module, we consider the results with the minimum RMSE, defined in (9), to be the optimal solution. All the algorithms are programmed and implemented in MATLAB environment to identify the PV module parameters using the single diode model on a standard PC with a 2.66 GHz Core i7 Intel CPU and 6 G RAM operating under the Windows 7 operating system.

### 4.1. Efficiency of MAFSA

**Figure 7:**
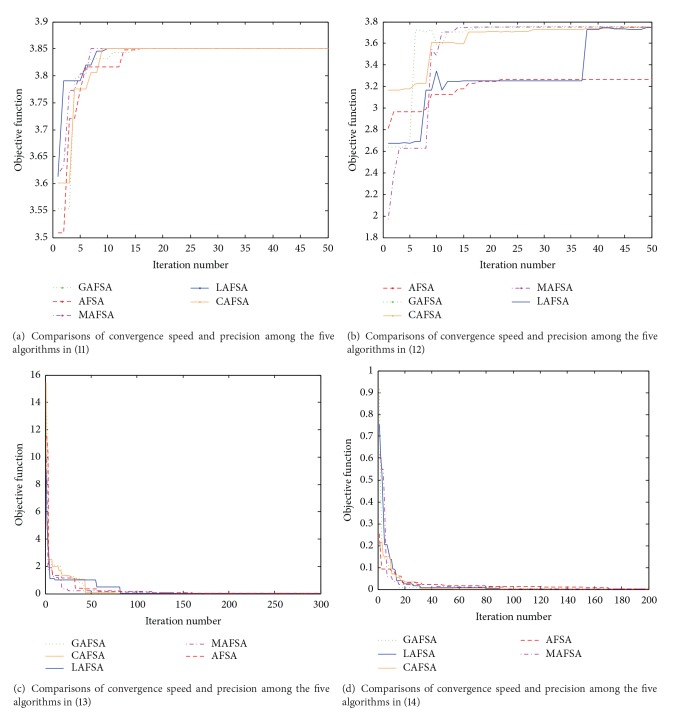
Comparisons of convergence speed and precision among the five algorithms in four benchmark test functions.

In order to study the performance of the proposed AFSA, four nonlinear functions have been used to investigate its optimization capacity, as shown in (11)–(14). All these functions are multimodal functions with a lot of local optima around global optima. When dealing with multimodal optimization problems, the standard AFSA is always stuck into local optima because of individual premature. Based on this, some mechanisms such as leaping behavior (LAFSA) [30], crossover operator (CAFSA) [31], and global information of AF (GAFSA) [32] have been proposed to improve the performance of the standard AFSA.  Figure 7 illustrates the comparisons of convergence speed and precision among the five algorithms.

The iteration times* MAXGEN* of four optimization functions are different according to the different function dimensions. The iteration times of (11)–(14) are 50, 50, 300, and 200, respectively. Similar simulation conditions, including* Sizepop*,* Step*,* Try_number*,* Visual,* and *δ*, are set to ensure a fair evaluation in the same benchmark test function as follows:* Sizepop* = 20,* Step* = 0.3,* Try_number* = 20,* Visual* = 1.5, and *δ* = 0.618. In addition, the probability of leaping behavior *β* and crossover operator *α* is selected the same as in [30, 31]

(11) $$\begin{array}{c} \max f\left(x\right)=x\sin\left(10\pi x\right)+2 \end{array}$$

subject to 1 ≤ *x* ≤ 2, where

(12) $$\begin{array}{c} \max f\left(x,y\right)=x\cos\left(2\pi y\right)+y\sin\left(2\pi x\right) \end{array}$$

subject to −2 ≤ *x*, *y* ≤ 2, where

(13) $$\begin{array}{c} \min f\left(x,y\right)=x^{2}+y^{2}-10\cos\left(2\pi x\right)-10\cos\left(2\pi y\right)+20 \end{array}$$

subject to −5 ≤ *x*, *y* ≤ 5, where

(14) $$\begin{array}{c} \min f\left(x,y\right)=\frac{1}{4000}\left(x^{2}+y^{2}\right)-\cos x\cos\left(\frac{y}{\sqrt{2}}\right)+1 \end{array}$$

subject to −10 ≤ *x*, *y* ≤ 10.

The simulation results demonstrate that four improved AFSAs can effectively enhance the convergence speed and optimizing precision by comparison with the standard AFSA. Meanwhile, the four improved AFSAs have the ability to avoid the problem of premature convergence. However, because the basic ideas of the four improved AFSAs are slightly different, the optimization effect may appear to have significant difference, especially in solving different optimization problems.

For LAFSA, the AFs gather around the extreme point in the later period of convergence, while the leaping step at this moment changes in a relative large range, so it does not avoid the problems of erratic fluctuation in the nearby of the optimal value. Therefore, it is difficult to obtain more accurate results of approximation by LAFSA.

For CAFSA, as evolution continues, the fish swarm tends to simplification. The effect of new individual AF generated by crossover operation might gradually disappear; the iteration process of AFSA will terminate soon. When the group size* Sizepop* is large, the calculated amount is inevitably increased and computational efficiency is also unavoidably influenced.

For GAFSA, when there exists an extraordinary local extremum (the fitness value of this local extremum is significantly bigger than others), this individual AF will be selected repeatedly with the action of adding global information in the position increment vector. Fish swarm in the following generation would be soon brought under control by the extraordinary individual AF, which results in low competitiveness of fish swarm. And the AFs may stay stuck in local extreme point easily.

Meanwhile, for MAFSA, the history of best AF is retained, and the other AFs mutate in a given probability. In this way, the best individual AF will not be lost in convergence process, and also the searching scope is enlarged. As a consequence, the MAFSA ensures population stability and diversity and increases the accuracy and convergence speed.

In comparison with the other improved AFSAs, it can be seen that the new AFSA with MO has no obvious deficiency and increases the possibility of searching the global optimum. Hence, it is feasible to identify the unknown parameters for PV module based on the AFSA with MO.

### 4.2. Identification Results for Experimental Data

**Figure 8:**
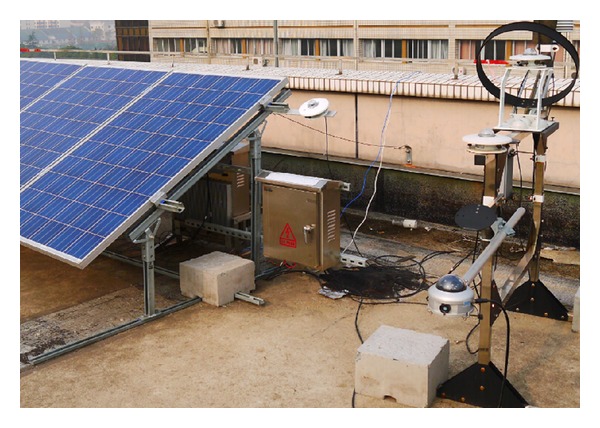
Outdoor test platform of PV modules on the roof.

In order to verify the effectiveness of proposed algorithm, several experiments were implemented on the established outdoor test platform on the roof of the Experiment Building in Hohai University Changzhou Campus (latitude 31.82 N, longitude 119.98 E) as shown in Figure 8. There are three PV modules installed onto the rack. A programmable electrical load controlled by digital signal processor (DSP) was designed to measure the* V*-*I* curves of PV modules [33]. The values of voltage and current are collected by high-precision voltage sensor TBV50AD and current sensor TBC10LX. The resolution of measured voltage and current is 10 mV in the range of 0–50 V and 1 mA in the range of 0–10 A, respectively. 30* V*-*I* points were measured for one* V*-*I* curve in approximately 20 ms, in case of weather condition changing. Coplane irradiance and the temperature of ambient temperature are measured simultaneously by the pyranometer TBQ-2 and temperature sensor Pt100, respectively. All the measured data were transmitted via a wireless local area network to the host PC in the laboratory.

The actual PV module temperature is relatively higher than the temperature of ambient temperature. So the following equation is used to transform PV module temperatures from measured ambient temperatures:

(15) $$\begin{array}{c} T=T_{\text{air}}+\frac{\text{NOCT}-20}{800}\times S, \end{array}$$

where *T* is PV module temperature, *T*_air_ represents the measured average temperature in the air, NOCT is the nominal operating cell temperature, and its value is approximately 48°C in general; *S* is the measured solar irradiance which is coplane with PV module. In this paper, one actual module was utilized, Trina TSM-250PC05A (poly-crystalline). There are 60 solar cells connected in series to form this type of PV module.

Meanwhile, it is assumed that the environmental conditions of all solar cells connected in one module are identical. The Newton-Raphson (NR) method in MATLAB software programmed by M-file is utilized for the parameters identification. For all the evolutionary algorithms, the population size* Sizepop* is set to 30 numbers and the maximum allowable generations* MAXGEN* are set to 100. For GA implementation, the crossover rate *P*_crossover_ = 0.4 and mutation rate *P*_mutation_ = 0.2 are utilized for the convergence process test. As for PSO implementation, the algorithm parameters are set as learning factors *c*_1_ = *c*_2_ = 1.49445, inertia weight factors *ω*_max⁡_ = 1 and *ω*_min⁡_ = 0.7, and velocity clamping factor *V*_max⁡_ = [2, 0.2, 0.4, 0.002, 2]. The parameters set for ABSO algorithm, obtained by trail, are as follows: the swarm size is set to 30 of which 25 bees are onlooker and 5 bees are scout, number of elites *n*_*e*_ = 5, radius of walk parameters *τ*_max⁡_ = 0.2 and *τ*_min⁡_ = 0.02, and decreasing linear parameters *ω*_*e*max⁡_ = *ω*_*b*max⁡_ = 2.5, *ω*_*e*min⁡_ = *ω*_*b*min⁡_ = 1.25. The parameters of MAFSA are consistent with the parameters in Section 4.1. It is worth noting that the initial setting parameter is obtained by multiple trials.

During the parameter identification process for the PV module, the values of the objective function in different optimization algorithms are shown in Table 2. Table 2 lists the model parameters of the Trina TSM-250PC05A (polycrystalline) PV module under irradiance condition 950 W/m^2^ at 25°C, which are identified from the experimental data. The optimal identified parameters along with the minimal RMSE values for single diode model found by the MAFSA are summarized, in comparison with the results obtained by other methods. As can be seen, it is obvious that the proposed algorithm outperforms the other ones since it has found the smaller RMSE values. For this reason, the optimal identified parameters found by the MAFSA are more close to the real PV module parameters than other methods.

**Figure 9:**
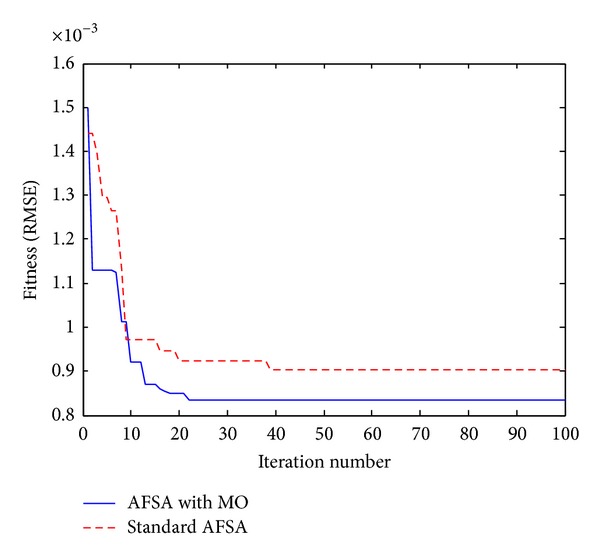
Convergence process of standard AFSA and AFSA with MO for parameters identification.

Moreover, in order to further evaluate the accuracy and effectiveness of the MAFSA-based parameters identification, the parameters identified by the standard AFSA and AFSA with MO are shown in Figure 9. To ensure the impartiality of comparisons, the stop criteria of both algorithms are equal to the maximum iteration number with 100. By comparison, the iterative precision of the standard AFSA is reached by 9.04*e* − 4 until the maximum iteration number is met, while the AFSA with MO is reached by 8.41*e* − 4 with only 22 iterative times. From the iteration process in Figure 9, it can be obviously seen that the AFSA with MO provides better results than the standard AFSA. The superiority of the MAFSA is reflected not only in convergence speed, but also in the convergence accuracy. This is due to the intelligent mutation operator employing unceasingly in the iteration process. Therefore, the improvement of the standard AFSA can obviously enhance the global search ability and achieve the desired results. The simulation results show the correctness and efficiency of the presented algorithm.

**Figure 10:**
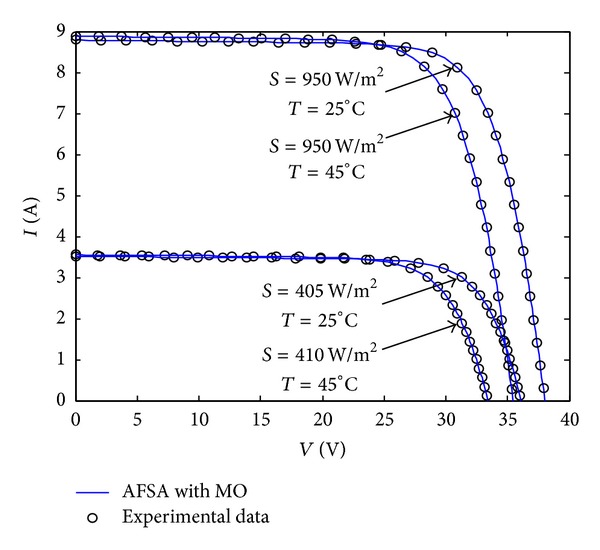
A comparison of* V*-*I* curves using the identified parameters from the AFSA with MO (solid lines) and the experimental data (dots) for TSM-250PC05A PV module under different irradiance and temperature conditions.

For further effectiveness of the proposed algorithm, the MAFSA is evaluated by using this type of PV module operating under different irradiance and temperature conditions. In this paper, four different irradiance conditions (*S* = 950 W/m^2^, 955 W/m^2^, 405 W/m^2^, 410 W/m^2^) but with different temperatures (*T* = 25°C, 45°C, 25°C, 45°C) are tested. After applying the proposed MAFSA to each experimental data, the identified parameter values and the minimal RMSE are illustrated in Table 3. Meanwhile, Figure 10 shows the simulated* V*-*I* curves using the identified parameters along with the experimentally measured* V*-*I* curves under different irradiance and temperature conditions.

As can be seen, the RMSE values of the testing PV modules are much lower, which indicates that the best objective function value can be obtained at each iteration process.

**Figure 11:**
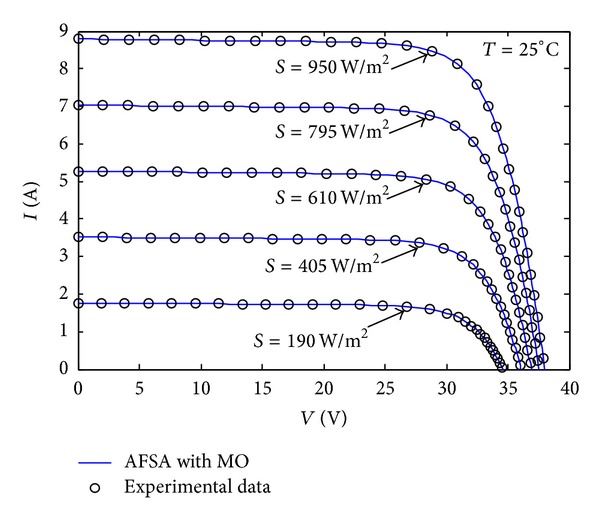
A comparison of* V*-*I* curves using the identified parameters from the AFSA with MO (solid lines) and the experimental data (dots) for TSM-250PC05A under five different irradiance conditions.

**Figure 12:**
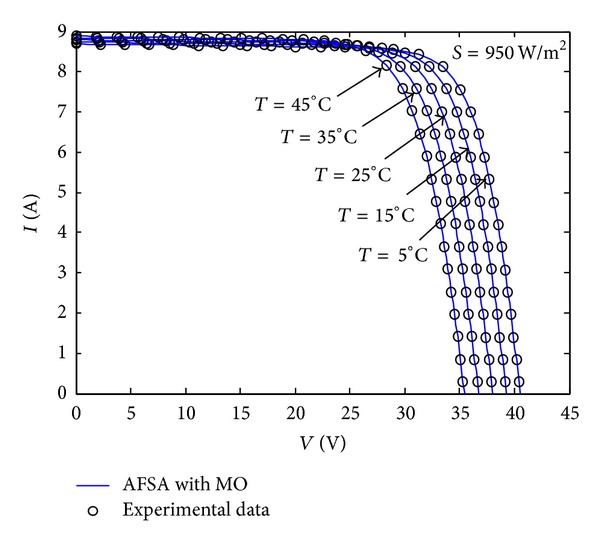
A comparison of* V*-*I* curves using the identified parameters from the AFSA with MO (solid lines) and the experimental data (dots) for TSM-250PC05A under five different temperature conditions.

In addition, the* V*-*I* characteristic curves generated using the parameters identified by the proposed method are plotted against the experimental data for the commercial PV module under different environmental conditions, namely, five different irradiances (*S* = 950 W/m^2^, 795 W/m^2^, 610 W/m^2^, 405 W/m^2^, 190 W/m^2^) with constant temperature (*T* = 25°C) and five different temperatures (*T* = 5°C, 15°C, 25°C, 35°C, 45°C) with constant irradiance (*S* = 950 W/m^2^). By the way, an intelligent temperature-control instrument is used to simulate temperatures in a large range. For the sake of clarity, we have only marked selected experimental data points, uniformly distributed within the* V*-*I* range, for each curve of TSM-250PC05A (polycrystalline) PV module in Figures 11 and 12, respectively. From Figures 11 and 12, we can see that the* V*-*I* values of the curves obtained using the identified parameters are quite consistent with the experimental data over the whole range, which means that the parameters identified by our proposed MAFSA can represent the intrinsic parameters of the PV module efficiency.

### 4.3. Computational Time and Parameters Identification Precision of the Proposed Method

Here, we report not only the number of function evaluations but also the computational time. The reason is that the computational time can reflect the efficiency of algorithms more comprehensively than the number of function evaluations in direct search methods.

However, how to fairly compare the GA, PSO, SA, and AFSA is a problem, as their mechanisms and parameters are different. Firstly, for all the evolutionary algorithms, the population size is set to 30 numbers and the maximum iteration number is set to 100 in Section 4.2. The individual code computing is 30 × 100 times for all evolutionary algorithms. For the sake of simplicity, the minimal computational time with simulation results from 10 runs for PV module is presented in Table 4.

From Table 4, it is very clearly seen that the computational efficiency of the MAFSA has been improved significantly compared with that of other methods.

In addition, to ensure the impartiality of comparisons, the stop criteria of all the evolutionary algorithms are equal to the iterative precision with 9.0*e* − 4. By constantly adjusting the parameters of the above algorithms, all the algorithms can always converge to the given iterative precision after several iterations. However, the difference is that each algorithm has the different selection of parameters, which lead to a great disparity of computational complexity among all the algorithms. The parameters of different algorithms are presented as follows.

1. For GA,* Sizepop* = 50,* MAXGEN* = 200, *P*_crossover_ = 0.6, and *P*_mutation_ = 0.1.
2. For PSO,* Sizepop* = 30,* MAXGEN* = 100, *c*_1_ = 2.7, *c*_2_ = 1.3, *ω*_max⁡_ = 0.9, and *ω*_min⁡_ = 0.3.
3. For ABSO,* Sizepop* = 50,* MAXGEN* = 500, *n*_*e*_ = 20, *τ*_max⁡_ = 0.2, *τ*_min⁡_ = 0.02, *ω*_*e*max⁡_ = *ω*_*b*max⁡_ = 2.0, and *ω*_*e*max⁡_ = *ω*_*b*max⁡_ = 1.0.
4. For AFSA,* Sizepop* = 50,* MAXGEN* = 100,* Step* = 0.1,* Try_number* = 50,* Visual* = 1.0, and *δ* = 0.618.
5. For MAFSA,* Sizepop* = 30,* MAXGEN* = 100,* Step* = 0.5,* Try_number* = 20,* Visual* = 2.5, *δ* = 0.618, and PMO = 0.05.

Obviously, we can see that the individual code computing for different algorithms is 50 × 200 times, 30 × 100 times, 50 × 500 times, 50 × 100 times, and 30 × 100 times, respectively. The computational time of the MAFSA is in direct proportion to the computational complexity. The corresponding computational times of the algorithms are 87 s, 64 s, 205 s, 77 s, and 53 s, respectively.

In the iterative processes, the PSO and ABSO for parameters identification have a large distribution of results, which cannot guarantee consistency in the identified solutions. Meanwhile, the MAFSA generated variation in a relatively small range and the standard deviation are small and tolerable. It is demonstrated that the proposed algorithm has a better quality of solution and robustness for parameters identification of PV module. In other words, the proposed method is able to effectively obtain the parameters of PV module and thus can always estimate the parameters with good accuracy and consistency.

## 5. Conclusion

This paper proposes parameters identification for PV module based on an improved AFSA. The feasibility of the proposed algorithm has been verified by identifying the parameters of one commercial PV module with single diode model under different operating conditions. The simulated current values are in good agreement with the experimental data, which mean that the proposed algorithm has high precision and fast convergence speed. Comparing with the other methods from the literatures, the results obtained by the MAFSA are quite superior and promising.

As a result, the improved AFSA algorithm is a useful way and can be efficiently applied to parameters identification for various types of PV modules. In future work, the fault diagnosis for PV modules will highlight the importance of this accurate parameters identification.

## Figures and Tables

**Algorithm 1:**
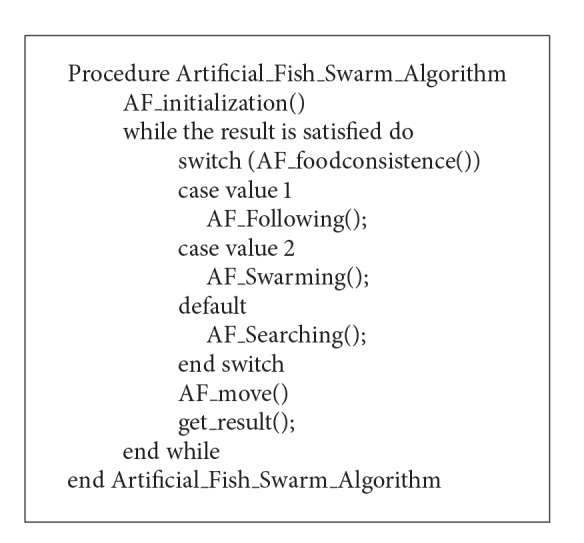
Procedure for the AFSA.

**Table 1:** Upper and lower ranges of the PV module parameters.

Parameter	Lower bound	Upper bound
*R*_*s*_ (Ω)	0	0.01
*R*_sh_ (Ω)	0	10
*I*_ph_ (A)	0	10
*I*_SD_ (*μ*A)	0	1
*n*	1	2

**Table 2:** Comparison of different methods for parameter identification with experimental data (TSM-250PC05A, *N*_*P*_ = 3, and *N*_*S*_ = 60).

Parameter	Method					
	NR	GA	PSO	ABSO	AFSA	MAFSA
*R*_*s*_ (Ω)	0.0047	0.0025	0.0021	0.0029	0.0028	0.0028
*R*_sh_ (Ω)	4.8973	4.9996	2.6191	4.9104	5.0017	5.0094
*I*_ph_ (A)	8.7984	8.7746	8.8808	8.7948	8.7950	8.7949
*I*_SD_ (*μ*A)	2.41*e* − 5	0.1044	0.8180	0.0922	0.1020	0.1021
*n*	0.9256	1.5123	1.5226	1.3416	1.3488	1.3488
RMSE	0.1517	9.98*e* − 4	1.17*e* − 3	1.51*e* − 3	9.04*e* − 4	8.41*e* − 4

**Table 3:** Parameters identification for the TSM-250PC05A PV module under different irradiance and temperature conditions (*S*: W/m^2^, *T*: °C, *N*_*P*_ = 3, and *N*_*S*_ = 60).

Parameter	TSM-250PC05A			
	S = 950	S = 955	S = 405	S = 410
	T = 25	T = 45	T = 25	T = 45
*R*_*s*_ (Ω)	0.0028	0.0028	0.0028	0.0028
*R*_sh_ (Ω)	4.9989	5.0020	6.6044	6.6075
*I*_ph_ (A)	8.7950	8.8889	3.5180	3.5556
*I*_SD_ (*μ*A)	0.1010	0.9470	0.1010	0.9470
*n*	1.3483	1.3417	1.3484	1.3417
RMSE	8.95*e* − 4	8.78*e* − 4	8.75*e* − 4	8.62*e* − 4

**Table 4:** Comparison of the computational time for parameters identification with other methods.

Parameter	Method				
	GA	PSO	ABSO	AFSA	MAFSA
Time/s	42	59	104	89	67
RMSE	9.81*e* − 4	9.12*e* − 4	1.36*e* − 3	8.98*e* − 4	8.41*e* − 4

## References

[1] Villalva MG, Gazoli JR, Filho ER (2009). Comprehensive approach to modeling and simulation of photovoltaic arrays. *IEEE Transactions on Power Electronics*.

[2] Carrero C, Ramírez D, Rodríguez J, Platero CA (2011). Accurate and fast convergence method for parameter estimation of PV generators based on three main points of the I-V curve. *Renewable Energy*.

[3] D'Alessandro V, Guerriero P, Daliento S, Gargiulo M (2011). A straightforward method to extract the shunt resistance of photovoltaic cells from current-voltage characteristics of mounted arrays. *Solid-State Electronics*.

[4] Zhu X, Fu Z, Long X (2011). Sensitivity analysis and more accurate solution of photovoltaic solar cell parameters. *Solar Energy*.

[5] Lo Brano V, Orioli A, Ciulla G, di Gangi A (2010). An improved five-parameter model for photovoltaic modules. *Solar Energy Materials and Solar Cells*.

[6] Phang JCH, Chan DSH, Phillips JR (1984). Accurate analytical method for the extraction of solar cell model parameters. *Electronics Letters*.

[7] Easwarakhanthan T, Bottin J, Bouhouch I, Boutrit C (1986). Nonlinear minimization algorithm for determining the solar cell parameters with microcomputers. *International Journal of Solar Energy*.

[8] Chan DSH, Phang JCH (1987). Analytical methods for the extraction of solar-cell single- and double-diode model parameters from I-V characteristics. *IEEE Transactions on Electron Devices*.

[9] Ortiz-Conde A, García Sánchez FJ, Muci J (2006). New method to extract the model parameters of solar cells from the explicit analytic solutions of their illuminated I-V characteristics. *Solar Energy Materials and Solar Cells*.

[10] Jain A, Kapoor A (2004). Exact analytical solutions of the parameters of real solar cells using Lambert W-function. *Solar Energy Materials and Solar Cells*.

[11] Jain A, Sharma S, Kapoor A (2006). Solar cell array parameters using Lambert W-function. *Solar Energy Materials and Solar Cells*.

[12] Jervase JA, Bourdoucen H, Al-Lawati A (2001). Solar cell parameter extraction using genetic algorithms. *Measurement Science and Technology*.

[13] Ye M, Wang X, Xu Y (2009). Parameter extraction of solar cells using particle swarm optimization. *Journal of Applied Physics*.

[14] Soon JJ, Low K (2012). Photovoltaic model identification using particle swarm optimization with inverse barrier constraint. *IEEE Transactions on Power Electronics*.

[15] AlHajri MF, El-Naggar KM, AlRashidi MR, Al-Othman AK (2012). Optimal extraction of solar cell parameters using pattern search. *Renewable Energy*.

[16] Askarzadeh A, Rezazadeh A (2012). Parameter identification for solar cell models using harmony search-based algorithms. *Solar Energy*.

[17] El-Naggar KM, AlRashidi MR, AlHajri MF, Al-Othman AK (2012). Simulated annealing algorithm for photovoltaic parameters identification. *Solar Energy*.

[18] Askarzadeh A, Rezazadeh A (2013). Artificial bee swarm optimization algorithm for parameters identification of solar cell models. *Applied Energy*.

[19] Li XL (2003). *A New Intelligent Optimization Method-Artificial Fish Swarm Algorithm*.

[20] Li XL, Lu F, Tian GH (2004). Applications of artificial fish school algorithm in combinatorial optimization problem. *Journal of Shandong University*.

[21] Shen W, Guo XP, Wu C (2011). Forecasting stock indices using radial basis function neural networks optimized by artificial fishswarm algorithm. *Knowledge-Based Systems*.

[22] Xu HY, Bai CL (2012). Study on application of local neighborhood algorithm for 40 Gb/s adaptive second-order polarization mode dispersion compensation. *Optical and Quantum Electronics*.

[23] Zhou YQ, Huang XS, Yang Y (2012). Hybrid optimization algorithm lased on mean particle swarm and artificial fish swarm. *Information-an International Interdisciplinary Journal*.

[24] Yu M, Liu D, de Dieu Bazimenyera J (2013). Diagnostic complexity of regional groundwater resources system based on time series fractal dimension and artificial fish swarm algorithm. *Water Resources Management*.

[25] Zhao W, Tang ZM, Yang YW, Wang L, Lan S (2014). Cooperative search and rescue with artificial fishes based on
fish-swarm algorithm for underwater wireless sensor networks. *The Scientific World Journal*.

[26] Ishaque K, Salam Z, Taheri H (2011). Simple, fast and accurate two-diode model for photovoltaic modules. *Solar Energy Materials and Solar Cells*.

[27] AlRashidi MR, AlHajri MF, El-Naggar KM, Al-Othman AK (2011). A new estimation approach for determining the I-V characteristics of solar cells. *Solar Energy*.

[28] Orioli A, di Gangi A (2013). A procedure to calculate the five-parameter model of crystalline silicon photovoltaic modules on the basis of the tabular performance data. *Applied Energy*.

[29] Sandrolini L, Artioli M, Reggiani U (2010). Numerical method for the extraction of photovoltaic module double-diode model parameters through cluster analysis. *Applied Energy*.

[30] Jiang M, Wang Y, Rubio F, Yuan D Spread spectrum code estimation by artificial fish swarm algorithm.

[31] Gao XZ, Wu Y, Zenger K, Huang X A knowledge-based Artificial Fish-swarm Algorithm.

[32] Jiang MY, Yuan DF, Cheng YM Improved artificial fish swarm algorithm.

[33] Ding K, Zhang JW, Bian XG, Xu JW (2014). A simplified model for photovoltaic modules based on improved translation equations. *Solar Energy*.

